# Transcutaneous trigeminal nerve stimulation modulates the hand blink reflex

**DOI:** 10.1038/s41598-020-78092-w

**Published:** 2020-12-03

**Authors:** Beniamina Mercante, Nicola Loi, Francesca Ginatempo, Monica Biggio, Andrea Manca, Ambra Bisio, Paolo Enrico, Marco Bove, Franca Deriu

**Affiliations:** 1 Department of Biomedical Sciences, University of Sassari, Viale San Pietro 43/b, 07100 Sassari, Italy; 2 Department of Experimental Medicine, Section of Human Physiology, University of Genoa, Genoa, Italy

**Keywords:** Physiology, Neurophysiology, Neuroscience, Cognitive neuroscience

## Abstract

The hand-blink reflex (HBR) is a subcortical response, elicited by the electrical stimulation of the median nerve, whose magnitude is specifically modulated according to the spatial properties of the defensive peripersonal space (DPPS) of the face. For these reasons, the HBR is commonly used as a model to assess the DPPS of the face. Little is known on the effects induced by the activation of cutaneous afferents from the face on the DPPS of the face. Therefore, we tested the effect of non-painful transcutaneous trigeminal nerve stimulation (TNS) on the amplitude of the HBR. Fifteen healthy participants underwent HBR recording before and after 20 min of sham- and real-TNS delivered bilaterally to the infraorbital nerve in two separate sessions. The HBR was recorded bilaterally from the orbicularis oculi muscles, following non-painful median nerve stimulation at the wrist. The HBR amplitude was assessed in the “hand‐far” and “hand‐near” conditions, relative to the hand position in respect to the face. The amplitudes of the hand-far and hand-near HBR were measured bilaterally before and after sham- and real-TNS. Real-TNS significantly reduced the magnitude of the HBR, while sham-TNS had no significant effect. The inhibitory effect of TNS was of similar extent on both the hand-far and hand-near components of the HBR, which suggests an action exerted mainly at brainstem level.

## Introduction

The peripersonal space, i.e. the space that directly surrounds us and which we directly interact with^1–3^, could serve diverse functions, some of which are linked with the need to protect ourselves from potential threats^4–6^. This protective function could trigger a specific type of peripersonal representation, namely the defensive peripersonal space (DPPS), intended as a vital “safety margin” surrounding the body^7–9^.

According to this view, potentially harmful stimuli occurring within the DPPS elicit stronger defensive responses, compared to stimuli located outside of it^4,7,10,11^. In this regard, a new reflex-based paradigm, the hand-blink reflex (HBR), has been described in humans following hand stimulation^12,13^. The HBR is a subcortical response whose magnitude is specifically modulated according to the spatial properties of the face DPPS, defining a high risk area where the reflex response is enhanced^3,4,14^. In particular, HBR amplitude increases more when the stimulated hand enters the subject’s DPPS around the face (near-HBR) than when the same stimulated hand is placed far from it (far-HBR). Further, HBR amplitude can be also modulated by internal factors that may influence the perception of the external stimuli, such as personality traits and cognitive expectations^7^, pain perception^3^, and acquired sensorimotor experience^15^.

It has been proposed that the HBR is mediated by fast subcortical pathways such as those underlying the somatosensory-evoked blink reflex (SBR), elicited by the electrical stimulation of the peripheral nerves of the limbs, namely the upper limb^16^ and the trigemino-facial blink reflex (TBR). The brainstem circuits mediating the HBR undergo top-down modulation from higher order cortical areas responsible for encoding the peripersonal space of the face, such as ventral intraparietal area (VIP) and the polysensory zone (PZ) in the precentral gyrus^13^. Interestingly, the DPPS of the head appears to have a privileged representation compared with the rest of the body^1^. A significant fraction of VIP neurons encoding the DPPS around the head receive visual-tactile information from the face^1,17,18^. In this regard, it is significant that in the monkey, air puffs directed to the face from a distance of 5 cm evoke a variety of stereotyped protective behaviors, similar to those elicited by the electrical stimulation of VIP and PZ^8^. These observations show that in terms of DPPS, different body parts may bear different value, and that the face could matter more than other body regions^7^. Thus, modulation of sensitive inputs from the face should affect any stereotyped defensive response related to it, such as the HBR. The somatosensory information necessary for the perception of changes in the external environment of the oral and facial regions is conveyed to the central nervous system (CNS) by the trigeminal nerve^19,20^. The trigeminal nerve projects primarily to the trigeminal nuclei in the brainstem, from where facial and oral sensory inputs are sent to the thalamus and then to the primary (S1) and secondary (S2) somatosensory cortices. The S1, in turn, sends back a contralateral projection to trigeminal nuclei and both S1 and S2 establish reciprocal monosynaptic excitatory connections with the motor cortex^21–24^ and other cortical areas such as the intraparietal sulcus, including the VIP^17,25,26^ . Besides its cortical projections, trigeminal nerve afferents influence the activity of brainstem neurons in the reticular formation, solitary tract nucleus, locus coeruleus and dorsal raphe nucleus which send extensive modulatory inputs to other subcortical and cortical areas^27^.

Owing to its extensive connections within the CNS, the trigeminal nerve is considered neuroanatomically strategic in influencing both cortical and subcortical structures, therefore it seems reasonable to hypothesize that the stimulation of trigeminal afferents may modulate the DPPS of the face. To answer this question, we investigated whether and how transcutaneous trigeminal nerve stimulation (TNS) modulates the HBR in healthy subjects. The TNS paradigm chosen to activate trigeminal afferents has been recently standardized in physiological^27–30^ and pathological conditions, such as neuropsychiatric disorders^31–34^, where TNS is widely used as a neuromodulation treatment method^35,36^.

## Results

Fifteen (7 females and 8 males; 24.26 ± 3.15 years old; range 21–30 years) out of thirty-one subjects (48%) showed a reproducible and stable HBR and were therefore included in the intervention phase and in the statistical analysis. None of the 15 subjects included in the study reported any side effects or pain apart from a state of relaxation during and/or after the real-TNS session.

Mean stimulus intensities for median nerve stimulation (real-TNS: 28.2 ± 5.93 mA, sham-TNS: 25.65 ± 3.66 mA; t_(14)_ = 0.637; *p* = 0.53) and threshold intensities for ION stimulation (real-TNS: 8.13 ± 0.25 mA for the left ION and 8.27 ± 0.34 for the right ION; t_(14)_ = − 0.397; *p* = 0.70; sham-TNS: 8.20 ± 0.31 mA for the left ION and 8.47 ± 0.37 for right the ION; t_(14)_ =  − 0.65; *p* = 0.52) were not different between the two experimental sessions.

**Figure 1:**
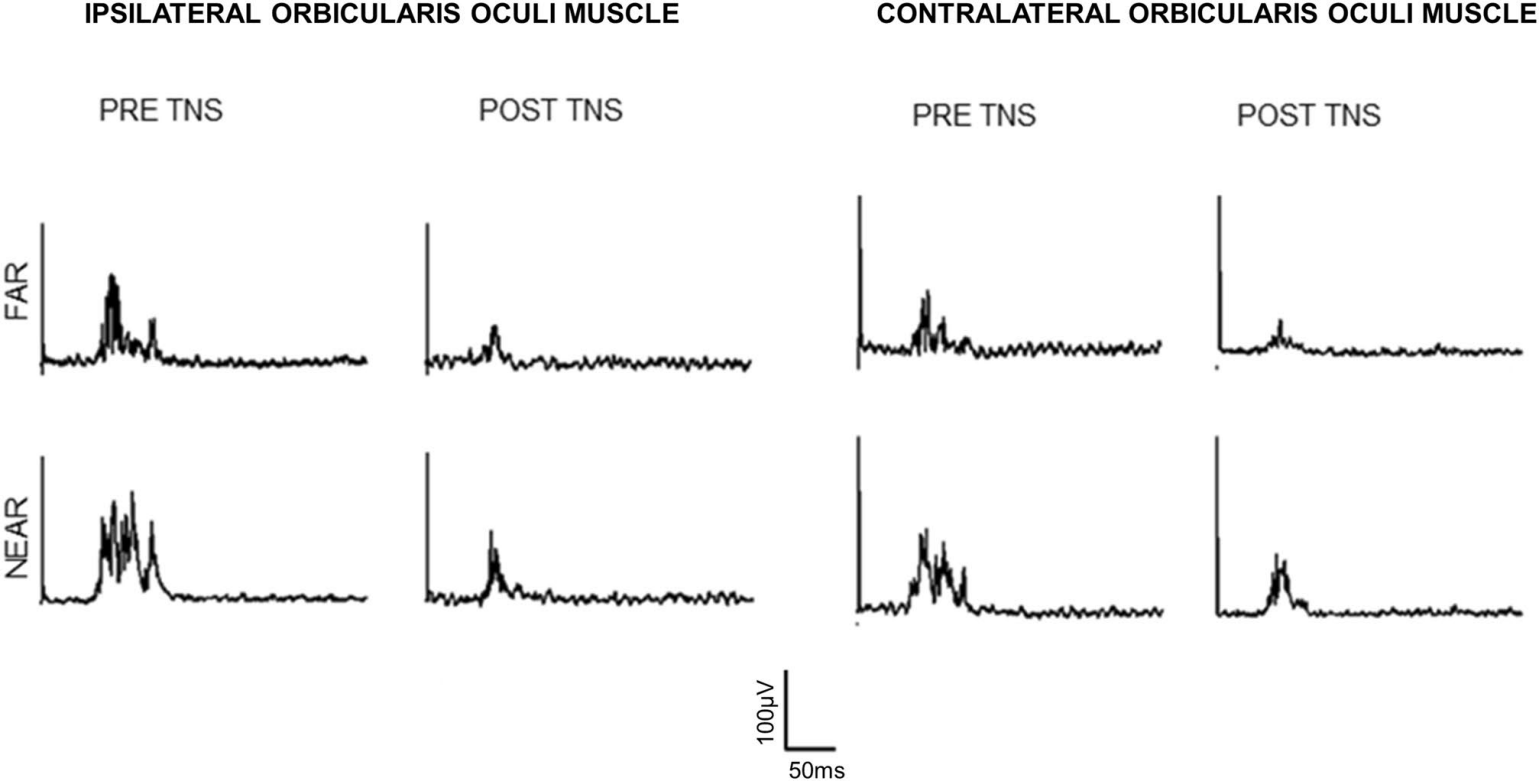
Recordings of Hand Blink Reflexes (HBR) from a representative subject before and immediately after delivery of real-TNS. The HBR was evoked by the electrical stimulation of the right median nerve in two different arm positions relative to the face (far and near).

**Figure 2:**
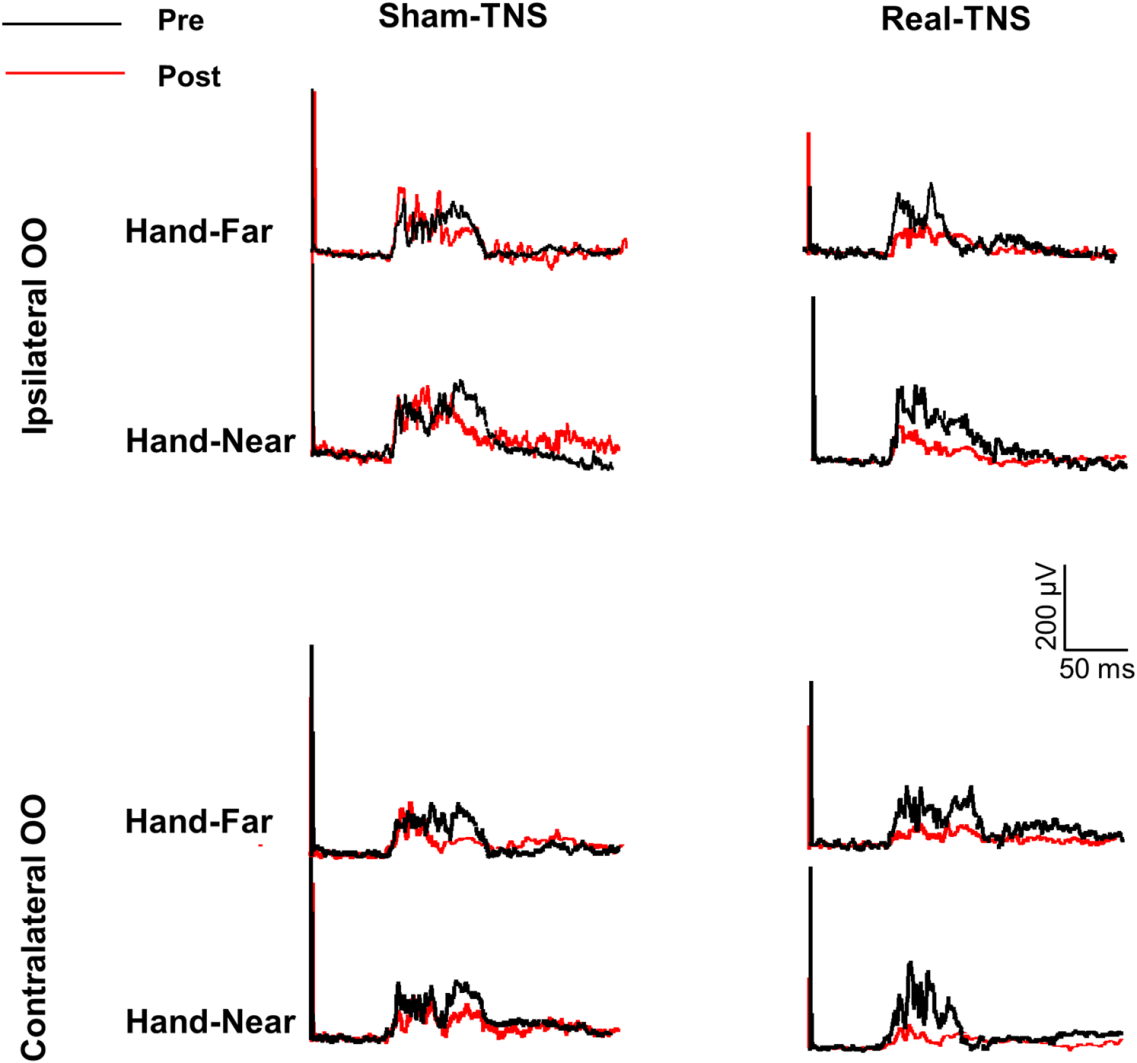
Gross average of mean Hand Blink Reflexes (HBR) traces recorded from all 15 subjects are shown by muscle (ipsilateral and contralateral Orbicularis Oculi muscle, OO), hand position in respect to the face (far-hand and near-hand) and experimental condition (sham- and real-TNS). The HBR recorded before TNS (PRE, black traces) and after TNS (POST, red traces) are superimposed. The artifacts of the stimuli indicate the application of the electrical stimulation of the right median nerve at the wrist.

Raw AUC values of the HBR before and after real- and sham-TNS are detailed in Table 1 by side of the orbicularis oculi muscle (OO) and hand position. Averaged responses recorded from a representative subject, before and after real-TNS, are shown in Fig. 1. The gross average obtained from mean traces of all subjects, recorded before and after sham and real-TNS, are shown in Fig. 2 by muscle (ipsi- and contralateral OO), hand position (far and near) and condition (sham- and real-TNS).

**Table 1:** Area under the curve of the hand-blink reflex before (pre) and after (post) short-term TNS.

Hand position	Orbicularis Oculi Muscle	Real-TNS		Sham-TNS	
		PRE	POST	PRE	POST
Far	Ipsilateral	37.57 ± 7.51	29.43 ± 5.37	35.34 ± 5.45	33.48 ± 4.51
	Contralateral	31.20 ± 6.09	25.32 ± 4.18	29.56 ± 4.41	28.04 ± 3.80
Near	Ipsilateral	44.16 ± 8.65	30.95 ± 5.51	43.91 ± 3.86	42.31 ± 3.22
	Contralateral	35.79 ± 6.47	26.89 ± 4.80	37.55 ± 4.51	35.39 ± 4.14

TNS, transcutaneous trigeminal nerve stimulation; values of the area under the curve are expressed in mV*ms and reported as mean ± standard error of the mean.

**Figure 3:**
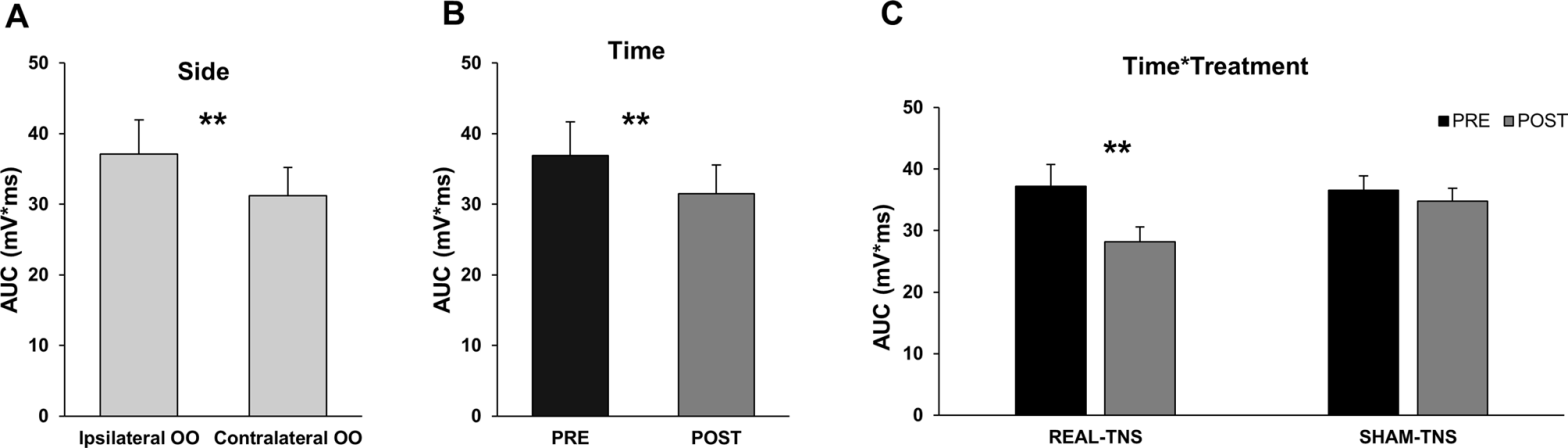
Hand Blink Reflexes (HBR) mean amplitudes (n = 15) are reported by (**A**) muscle side (ipsi- and contralateral orbicularis oculi muscles, OO), (**B)** time (PRE and POST treatment) and (**C**) time*treatment interaction. The amplitude of the HBR, expressed as area under the curve (AUC) ± standard error of the mean, was significantly reduced following amplitude of the HBR. *p < 0.05.

Data showed that the HBR amplitude was larger in the ipsilateral OO than in the contralateral OO (Fig. 3A) and in the near-hand than far-hand position. In particular, a four-way RM-ANOVA showed a significant effect of hand position (F_1,14_ = 4.791, *p* = 0.04), side (F_1,14_ = 11.841, *p* = 0.004), time (F_1,14_ = 14.025, *p* = 0.002) but a non-significant effect of treatment (F_1,14_ = 0.256, *p* = 0.621). Moreover, the analysis showed a non-significant effect of all the interactions among the factors except for the treatment x time (F_1,14_ = 5.767, *p* = 0.031) Figs. 3B,C. The post-hoc analysis of the interaction treatment x time showed that real and sham-TNS were non-significantly different at baseline (t_(14)_ = − 0.082; *p* = 0.936) and that responses were significantly smaller in the POST than in the PRE following real-TNS (t_(14)_ = 3.383; *p* = 0.004) but not following sham-TNS (t_(14)_ = 1.414; *p* = 0.179).

## Discussion

Short-term TNS was able to depress the amplitude of the HBR not only when the stimulated hand was located outside the DPPS surrounding the face (hand-far condition) but also when it was inside it (hand-near condition), whereas sham stimulation had no significant effects in both conditions.

The most likely mechanism responsible for the observed inhibitory effects may be a direct action of TNS on brainstem circuits mediating the HBR. These circuits consist of a double set of premotor interneurons, each underlying its hand-far or hand-near components. The first interneuronal pool is a common relay station for the hand-far component of the HBR (corresponding to the SBR) and the R2 component of the TBR. These interneurons are responsive to multiple sensory impulses (somatosensory, auditory, photic, etc.)^37^, which might inhibit each other, possibly at presynaptic level, leading to the so called “gating by presynaptic inhibition”. The activity of OO motoneurons is influenced by peripheral somatic inputs through at least two different reflex mechanisms: one is facilitatory (the SBR) and the other is inhibitory (the exteroceptive suppression)^16^. Common blink premotor interneurons in the brainstem reticular formation^16,38^ receive these multimodal sensory inputs and relay them through polysynaptic pathways to the OO motoneurons shaping their motor output. The depression of the hand-far HBR following real-TNS is congruent with previous data showing a similar effect on the R2 component of the TBR^28,30^.

The second pool of brainstem interneurons, which may be the target of TNS inhibitory action, are those mediating the hand-near component of the HBR. These interneurons undergo a top-down regulation exerted by PZ and VIP areas, which have been suggested to encode and modulate the defensive behavior within the DPPS^17,39–41^. Such modulation is heterosegmentally specific for the brainstem interneurons mediating the HBR^12^, which are thought to be different from those mediating the TBR. It has been reported that their level of excitability is fine-tuned as a function of the probability of stimulus recurrence^13^ by the PZ, VIP, premotor and parietal cortices, ventral premotor cortex and posterior parietal cortex^42^. The TNS inhibitory effect on these specific premotor neurons may be exerted directly or indirectly through an action on the brainstem reticular formation, which might act as an integrator of both excitatory and inhibitory inputs in the presence of various supranuclear influences^43^.

In summary, the TNS-induced decrease of the HBR might result from a TNS-induced decrease of the response of brainstem interneurons to median nerve stimulation. The activation of trigeminal afferents could reduce the excitability of these HBR premotor neurons, with a consequent decrease of the signal transmission to OO motoneurons, resulting in a decreased HBR response.

In our study, the baseline amplitude of the HBR was significantly larger in the hand-near than hand- far condition. This finding is in agreement with the literature on the topic, showing that the amplitude of the HBR increases consistently when the stimulated hand enters the face DPPS^12,13^.

**Figure 4:**
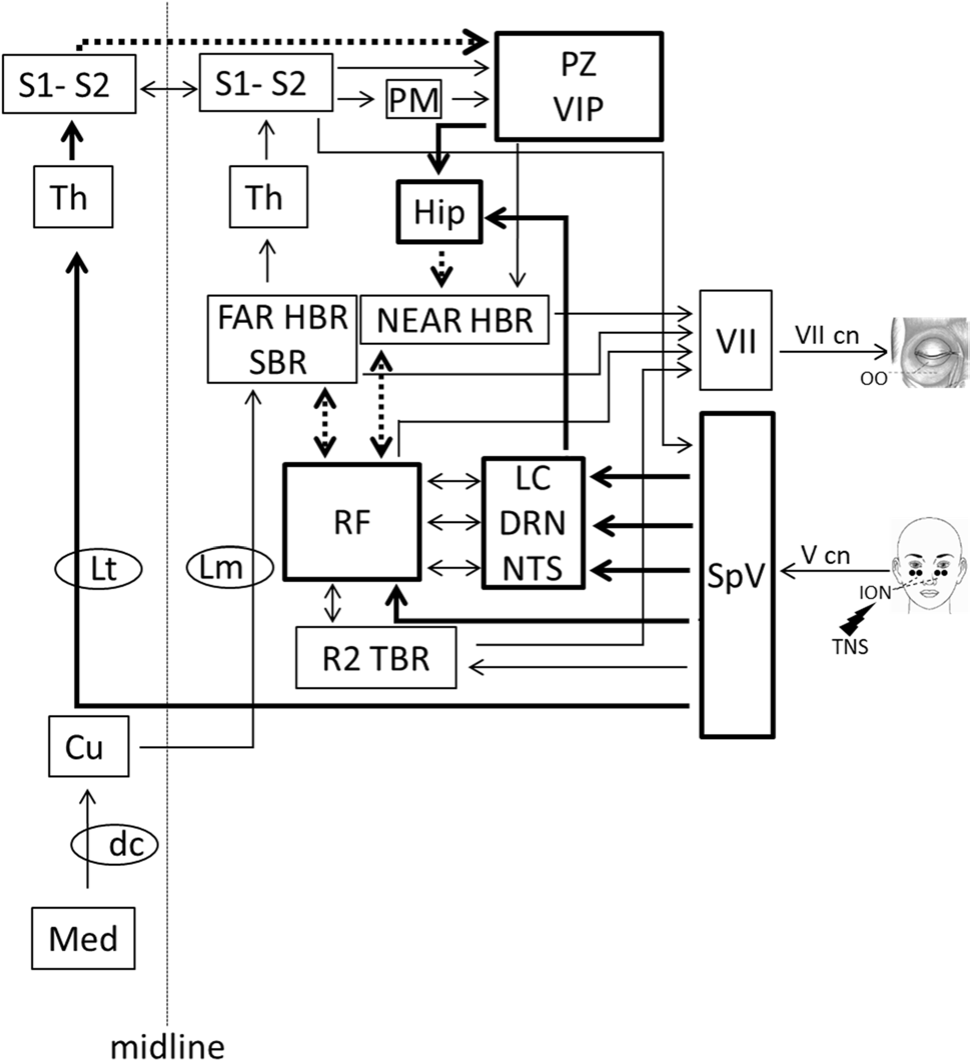
Overview of the possible pathways and structures involved in the neuromodulatory effect exerted by TNS on the neural circuits mediating the far- and near-hand blink reflex. In the figure the circuits mediating the spinal-blink reflex (SBR), the far-hand blink reflex (FAR HBR), the near-hand blink reflex (NEAR HBR) and the R2 component of the trigeminal blink reflex (R2 TBR) are schematically represented. Thick arrows indicate the pathways and thick boxes the structures possibly engaged by TNS and dotted lines represent the hypothesized inhibitory projections. Abbreviations: Cu: cuneatus nucleus; dc: dorsal column; DRN: dorsal raphe nucleus; FAR HBR: interneurons involved in the far-hand blink reflex; Hip: hippocampus; ION: infraorbital nerve; LC: locus coeruleus; Lm: lemniscus medialis; Lt: lemniscus trigeminalis; Med: median nerve; NEAR HBR: interneurons involved in the near-hand blink reflex; NTS: nucleus of the solitary tract; OO: orbicularis oculi muscle; PM: premotor cortex; PZ: polisensory zone; R2 TBR: interneurons mediating the R2 response of the trigemino-blink reflex; RF: reticular formation; S1: primary somatosensory cortex; S2: secondary somatosensory cortex; SBR: spinal-blink reflex; SpV: spinal trigeminal nucleus; Th: thalamus; TNS: transcutaneous trigeminal nerve stimulation; V cn: trigeminal nerve; VII cn: facial nerve; VII: facial motor nucleus; VIP: ventral interparietal area.

The HBR magnitude is finely tuned depending on the estimated probability that the threatening stimulus will occur, as well as on the presence of defensive objects near the face. The enhancement of the HBR response when the hand enters the DPPS is due to a cortico-bulbar facilitation of the polysynaptic medullary pathways that relay somatosensory inputs to the heterosegmentally specific HBR interneurons in the brainstem^12^. The power of this facilitation is determined by cognitive factors, which demonstrates the behavioral relevance of this subcortical reflex and how such fine top-down modulation is expression of its defensive value^12,15^. In our study, the TNS depressive action on the HBR was not significantly larger in the near (29.9%) than far (21.7%) hand positions of the reflex. This finding indicates as unlikely a TNS action on the cortical neurons involved in sensorimotor integration of polysensory information from the face DPPS, and in generation of appropriate behavioral and motor responses (such as the hand-near HBR). However, this hypothesis cannot be completely excluded since, besides S1 and S2, trigeminal information is conveyed to multiple cortical areas such as the intraparietal sulcus, including the VIP^17,25,26^, orbital cortex, perirhinal cortex and the entorhinal cortex^44^, which have been shown to be a target for TNS^27^. So, even if our experiments were not able to suggest a TNS action at cortical level, it may deserve further investigation in a larger number of subjects to compensate for the large variability of the reflex and increasing the spatial distance between the hand and the face in the hand-far condition to better separate the two components of the HBR. An overview of the possible sites of TNS action on the HBR is outlined in Fig. 4.

Interestingly, in patients with trigeminal neuralgia, the HBR is increased on the affected side^3^, suggesting that in this condition the DPPS is larger. This observation is apparently in conflict with our results, showing that after TNS, the HBR amplitude is consistently reduced. However, the two conditions cannot be compared, being the TNS used in our study a non-painful, short-term stimulation, while the former condition is characterized by a chronic hyperalgesia state. In this condition, because of their potential to evoke a painful sensation, trigeminal stimuli could modify the spatial properties of the face DPPS by increasing it in response to a continuous alarm^3^. Other conditions, such as the personality trait, the psychic state, threat and the cognitive expectations of the subject may modulate the amplitude of HBR^7,14,15^. Interestingly, the locus coeruleus, which is a target for TNS^27^, can modulate the occurrence of blinking, depending on the arousal state, emotions or alertness^45^. In addition, inhibitory actions exerted by multiple modalities of sensory stimulation as well as by supranuclear influences (cerebellum, basal ganglia, cerebral cortex), which are related to the sensory gating mechanism responsible for the interruption of the excessive inflow of somatosensory stimuli^16^, may influence not only the amplitude of the reflex, but also its occurrence. Therefore, the brainstem component of the reflex “may not normally be present because it has been habituated or inhibited”^16^. Although these hypotheses may explain why the frequency rate of the HBR is variable in physiological conditions (48% in our study, from 42.8 to 60% in other studies)^13,16,46^, it should be noted that a systematic investigation on the central and peripheral factors which can affect the occurrence of the HBR has never been performed. Depending on the context, these numerous factors may eventually drive the motor command towards opposite responses, i.e. closing the eye or keeping it open^16^.

Taken together, our data suggest that non-painful, short-term TNS significantly decreases the amplitude of the HBR in healthy subjects. This action is of similar extent on both the hand-far and hand-near components of the reflex, which suggests an action exerted mainly at brainstem level. This hypothesis fits with previous findings demonstrating that the TNS protocol here used depresses significantly the R2 component of the TBR, with a long-term depression-like mechanism^30^. Another possible mechanism could be a decrease in vigilance and arousal during and immediately after TNS, which has been previously described^47,48^ and observed, accordingly, in our subjects after the real-TNS session.

In the perspective of the functional meaning of the HBR, the TNS-inhibitory action on this protective reflex can appear as disadvantageous, since by dampening the response, the boundaries of the face DPPS would be reduced. However, this effect might also be viewed as functionally advantageous. In fact, if the face DPPS is reduced, the stimulated hand is no longer positioned inside its borders (i.e., near), but outside them (i.e., far), thus becoming a harmless stimulus from which the subject could distract his/her attention and engage it in perceiving more relevant stimuli coming from the surrounding environment.

## Materials and methods

### Participants

Thirty-one right-handed volunteers (11 females and 20 males; 24.38 ± 3.13 years old; range 20–32 years) were enrolled. A written informed consent was obtained from all subjects prior to study entry. All experimental procedures were approved by the local ethics committee (Azienda Sanitaria Locale 1 Sassari, Italy, number 2075/CE) and conducted in accordance with the Helsinki Declaration. None of the participants had history and/or current signs/symptoms of neurological and/or psychiatric diseases. Experiments were performed in a quiet room. Subjects sat in a comfortable chair and were asked to keep their eyes opened and to remain relaxed but alert during the experiments.

**Figure 5:**
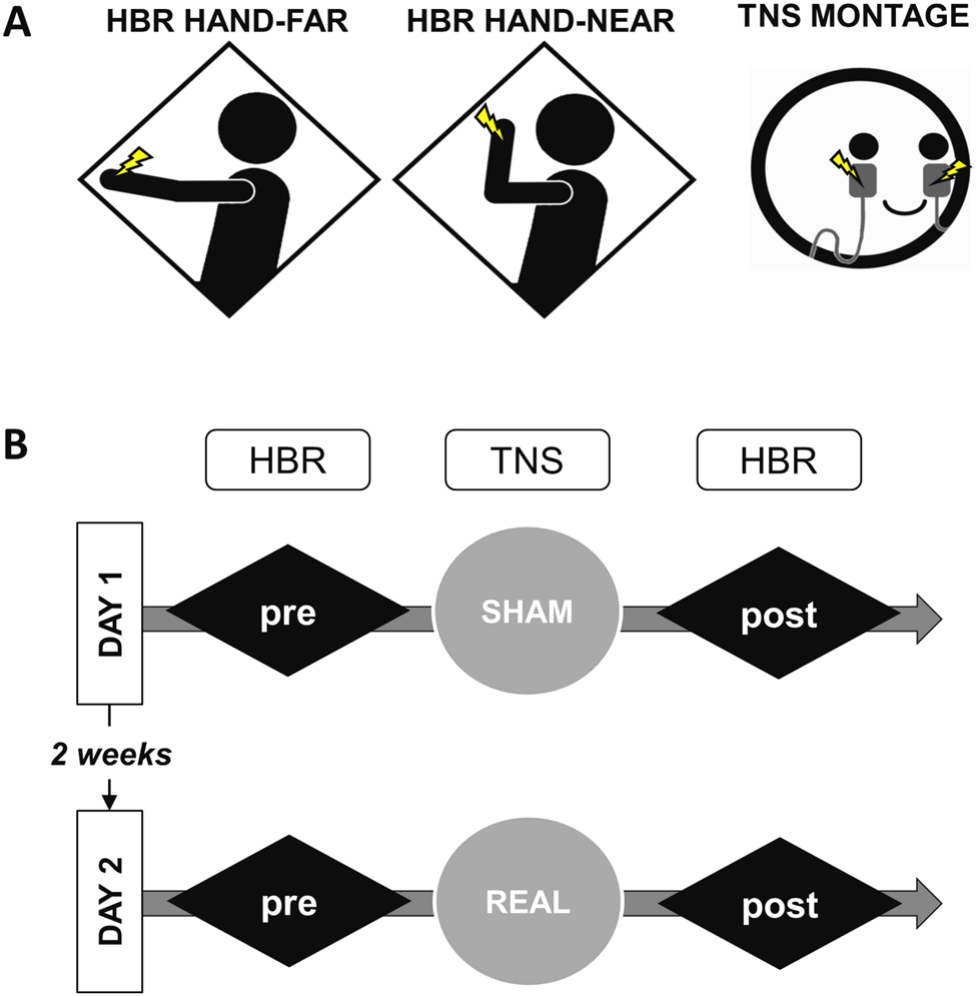
Experimental set-up. **A.** Set-up for the recording of the hand-blink reflex (HBR) in the hand-far and hand-near positions and electrode montage used for the bilateral transcutaneous trigeminal nerve stimulation (TNS). The yellow lightning bolts indicate the electrical stimulation of the right median nerve at the wrist in the HBR recording set-up and the electrical stimulation of the infraorbital nerve bilaterally, both at intensities below the pain threshold. **B.** Experimental protocol. The HBR was recorded in the two hand positions randomly before (pre) and after (post) TNS. The sham and real TNS were performed at least two weeks apart.

Each volunteer underwent a sham TNS session first and a real TNS session afterwards. Sessions were separated by at least 2 weeks to avoid possible confounding after‐effects. During each session, the HBR was assessed at baseline and immediately after TNS. HBR was recorded in two discrete experimental conditions, characterized by different arm positions relative to the face (far and near) known to alter HBR^49^. The experimental set-up is depicted in Fig. 5.

### Electromyography (EMG)

Surface EMG was recorded (D360 amplifier; Digitimer Ltd, Welwyn Garden City, UK) bilaterally from the OO. The active electrode was placed over the lower lid, the reference electrode 2 cm far from the lateral cantus and the ground electrode over the right part of the forehead. Signals were filtered (band pass 5–5000 Hz), amplified and digitized at a sampling rate of 8196 Hz through a CED1401 power analog-to-digital converter (Cambridge Electronic Design, Cambridge, UK). Signal 5.0 software was used for data collection and offline analysis^28,30^.

### Hand-blink reflex (HBR)

The HBR response was elicited by delivering transcutaneous electrical stimuli to the median nerve at the right wrist via cup electrodes connected to a DS7A Stimulator (Digitimer, Welwyn Garden City, Herts, UK). All stimuli were square waves (0.2 ms duration and at variable time intervals between 20 and 40 s to minimize habituation of the HBR). Stimulus intensity (range 8–72 mA) was always subthreshold for pain and was adjusted to elicit in each participant a clear and stable HBR in three consecutive trials. Subjects who did not meet this criterion or did not exhibit any response at median nerve stimulation intensity below the pain threshold were excluded from the study.

Electrical stimulation of the median nerve was delivered in static conditions, while participant’s right hand was located at two different positions relative to the face, as described by Farnè et al.^49^. More in detail, in the first position (hand‐far), participant’s forearm was resting on a table with the elbow joint flexed at 120°. In such positioning, the wrist resulted at a distance of 60 cm from the ipsilateral side of the face and the hand was below the lower limit of the visual field. In the second position (hand‐near), participant’s elbow was flexed at 75° with respect to the arm, with the wrist at a distance of 4 cm from the ipsilateral side of the face. These positions caused the hand being seen as outside (far condition) or inside (near condition) the DPPS of the face^49^. Throughout the experiment, participants were instructed to keep their gaze on a fixation point placed at 60 cm in front of their eyes. The left arm was never stimulated, and was held along the body throughout the duration of the experiment. Participants were instructed, trial by trial, to put the arm in one of the two positions previously identified. Twenty acquisitions were performed, 10 for each hand position. The order of the hand positions at which the participant received the electrical stimulus was random.

### Transcutaneous trigeminal nerve stimulation (TNS)

Real-TNS was delivered bilaterally to the infraorbital nerve (ION) using 26 mm-diameter disposable, silver-gel, self-adhesive stimulating electrodes (Globus, Domino s.r.l., Codognè, TV, IT) positioned over the ION foramina and connected to a Winner stimulator (Fisioline biomedical instrumentation, Verduno, CN, IT)^50^. In the real-TNS, the stimulus consisted of trains of a symmetric biphasic square wave pulse of 0.25 ms duration and 120 Hz frequency. The stimulation was delivered in a cyclic modality (30 s ON and 30 s OFF) for a period 20 min, according to previous works^28,30,50^. Stimulation intensities ranged from 6 to 18 mA and corresponded, for each ION, to the highest pain sub-threshold intensity endurable comfortably by the subject.

The sham-TNS protocol mimicked the initial bilateral real-TNS stimulus perception and consisted of a previous calculation of both perceptual and pain threshold, followed by 20 s of TNS, the intensity of which induces initial skin sensations indistinguishable from real-TNS. The stimulation intensity was subsequently gradually decreased down to zero, which corresponded to the OFF position of the stimulator. Participants were not aware of the type of stimulation administered. In particular, they were aware they would have received two different types of intervention but were not aware that one of these was a sham. The two weeks elapsed between the two interventions allowed not only to avoid after-effects but also to minimize any memory related to the intervention-associated perception.

Blinding of the outcome assessor and statistician was obtained by labeling for the type of intervention with non-identifying terms (A and B), randomly assigned to sham and real-TNS protocols and only personnel delivering TNS were aware of the treatment allocation. No communication occurred among personnel, outcome assessor and statistician regarding trial course and participants.

### Data processing and statistical analysis

Based on previous studies^16,46^, we anticipated a detection rate of HBR of approximately 50%. Therefore, at least 31 subjects had to be tested for HBR presence to prospectively obtain complete data from 15 subjects.

EMG recordings from OO muscles were averaged separately in the hand-near and hand-far conditions. The area under the curve (AUC) of the HBR in each condition and side were measured within a 130 ms time interval from the stimulus onset that always contained the blink response. The resulting curve was then integrated to compute AUC. Statistical analysis was performed with SPSS 20 software (SPSS Inc, Chicago, IL, USA). Student’s paired t-test, repeated measures (RM) analysis of variance (ANOVA) and planned post hoc t-test with Bonferroni correction for multiple comparison were used. Compound symmetry was evaluated with the Mauchly’s test and the Greenhouse–Geisser correction was used when required. Significance was set for *p* value < 0.05. Values are expressed as means ± standard error of the mean (SEM).

The threshold intensity for median nerve stimulation as well as for ION stimulation were compared between real and sham sessions using paired t-test. In each participant, the AUC of the HBR trace was measured for each hand position, recording side, time and treatment. A four-way RM-ANOVA was used with treatment (real-TNS and sham-TNS), side (ipsilateral and contralateral), hand position (far and near) and time (PRE and POST), as within factors. In case of significant values, Student’s paired t test was used for post hoc analysis applying the Bonferroni correction for multiple comparisons.

## Data Availability

The datasets generated during and/or analysed during the current study are available from the corresponding author on reasonable request.
